# A Novobiocin Derivative, XN4, Inhibits the Proliferation of Chronic Myeloid Leukemia Cells by Inducing Oxidative DNA Damage

**DOI:** 10.1371/journal.pone.0123314

**Published:** 2015-04-30

**Authors:** Lixian Wu, Xianling Chen, Lisen Huang, Jue Tian, Fang Ke, Jianhua Xu, Yuanzhong Chen, Ming Zheng

**Affiliations:** 1 Department of Pharmacology, School of Pharmacy, Fujian Medical University (FMU), Fuzhou, P. R.China; 2 Institute of Materia Medica, FMU, Fuzhou, P. R.China; 3 Fuijan Key Laboratory of Natural Medicine pharmacology, FMU, Fuzhou, P. R.China; 4 Department of Pharmacochemistry, School of Pharmacy, FMU, Fuzhou, P. R.China; 5 Fujian Institute of Hematology, Union Hospital, FMU, Fuzhou, P. R.China; 6 Department of Anatomy, School of Basic Medicine, FMU, Fuzhou, P. R.China; B.C. Cancer Agency, CANADA

## Abstract

XN4 might induce DNA damage and apoptotic cell death through reactive oxygen species (ROS). The inhibition of proliferation of K562 and K562/G01 cells was measured by MTT (3-(4,5-Dimethylthiazol-2-yl)-2,5-Diphenyltetrazolium Bromide). The mRNA levels of NADPH oxidase 1-5 (*Nox1-5*) genes were evaluated by qRT-PCR. The levels of extracellular reactive oxygen species (ROS), DNA damage, apoptosis, and cell cycle progression were examined by flow cytometry (FCM). Protein levels were analyzed by immunoblotting. XN4 significantly inhibited the proliferation of K562 and K562/G01 cells, with IC_50_ values of 3.75±0.07 µM and 2.63±0.43 µM, respectively. XN4 significantly increased the levels of *Nox4* and *Nox5* mRNA, stimulating the generation of intracellular ROS, inducing DNA damage and activating ATM-γ-H2AX signaling, which increased the number of cells in the S and G2/M phase of the cell cycle. Subsequently, XN4 induced apoptotic cell death by activating caspase-3 and PARP. Moreover, the above effects were all reversed by the ROS scavenger *N*-acetylcysteine (NAC). Additionally, XN4 can induce apoptosis in progenitor/stem cells isolated from CML patients’ bone marrow. In conclusion, XN4-induced DNA damage and cell apoptosis in CML cells is mediated by the generation of ROS.

## Introduction

Reactive oxygen species (ROS), such as hydrogen peroxide, hydroxyl radicals, and superoxide anions, are highly reactive molecules with unpaired electrons; they are generated in physiological processes that mainly depend on the Nox enzyme, which catalyzes the production of NADP^+^ and O_2_^2-^ from NADPH. Intracellular ROS not only play an important role in signal transduction but also induce damage to cell structure when ROS generation exceeds the cellular antioxidant defense [1]. Cellular defenses against ROS include antioxidant scavengers and antioxidant enzymes. The excessive production of ROS has been shown to cause cell death by DNA-damage-induced cell cycle arrest in several types of cancer cells [2–6]. ROS production also decreases the mitochondrial transmembrane potential (ΔΨ_m_), in addition to the release of cytochrome *c*, and leads to mitochondrial-dependent apoptosis in a variety of human cancer cells [7–8]. Thus, the generation of ROS can be exploited therapeutically in the treatment of cancer.

The Bcr-Abl kinase inhibitor imatinib is a standard treatment for Ph+ chronic myeloid leukemia (CML) and has been shown to induce a complete hematologic and cytogenetic response in most chronic-phase CML patients. Despite outstanding clinical results, imatinib-resistant leukemia and clinical relapse eventually emerge. The mechanisms of resistance to imatinib include mutations of the Bcr-Abl kinase domain, the amplification of the *bcr-abl* gene, and the accumulation of chemotherapy-resistant leukemia stem cells [9–12]. Thus, it is critical to develop a novel approach beyond Bcr-Abl targets to overcome imatinib resistance.

**Fig 1:**
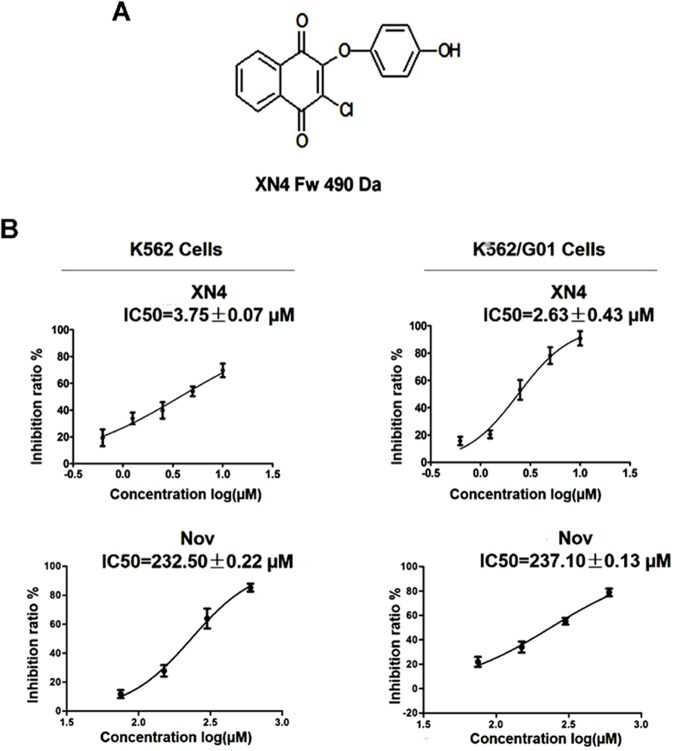
The proliferation inhibition effects of XN4 in K562 and K562/G01 cells. (A) The chemical structure of XN4. The molecular weight of XN4 is 490 Da. (B) K562 and K562/G01 cells were incubated with XN4 or Nov for 48 h. The cell viability was evaluated by MTT. The data were plotted as a function of percent cell viability based on controls vs. drug concentrations. All studies were performed in triplicate. The concentration of drug at which cell growth was inhibited by 50% (IC_50_) was estimated using the GraphPad Prism5 software.

Novobiocin (Nov) is a member of the coumermycin family of antibiotics and is a well-established inhibitor of DNA gyrase. Our previous studies have shown that Nov binds to the C-terminus of Hsp90 and induces the degradation of the Hsp90-dependent client protein, Bcr-Abl, with low activity. The Nov IC_50_ in K562 cells can reach 500 μM [13]. In an effort to develop more efficacious anti-cancer agents, our group synthesized a series of Nov derivatives; XN4 is one of the most active agents (Fig 1A). Herein, we show that XN4 has approximately 100-fold higher activity than its parental compound. XN4 was able to induce DNA damage by generating ROS, resulting in apoptotic cell death and cell cycle arrest in imatinib sensitive- and resistant- CML cells. Moreover, XN4 induces apoptosis in CD34+CD38- and CD34+CD38+ cells isolated from CML patients’ bone marrow.

## Materials and Methods

### Cell lines and Reagents

Human leukemic K562 cells were obtained from the Cell Bank of the Chinese Academy of Sciences (Shanghai, China). These cells were cultured and passaged in RPMI 1640 containing 10% heat-inactivated fetal bovine serum, 100 U/ml penicillin, 100 mg/ml streptomycin, and 2 mM glutamine (medium A) in a 5% humidified CO_2_ atmosphere at 37°C. Imatinib-resistant K562/G01 cells were purchased from the Institute of Hematology, Chinese Academy of Medical Sciences and Peking Union Medical College, Tianjin, China. The K562/G01 cells were maintained in medium A either containing or lacking 4 μM imatinib. XN4 was synthesized by our lab with 98% purity. It was resolved in dimethylsulfoxide (DMSO) in order to obtain a stock solution with a concentration of 10 mM. In all the experiments, the control cells were incubated with DMSO alone. The final concentration of DMSO was maintained at 0.1% w/v. MTT (3-(4, 5-Dimethylthiazol-2-yl)-2,5-diphenyltetrazolium bromide, a yellow tetrazole) were purchased from Sigma-Aldrich, Inc (MO, USA). Monoclonal antibodys of PARP，cleaved-caspase3, r-H2AX (pS139), p-ATM (ser 1981)，CDK2, β-actin were all purchased from Cell Signaling Technology, Inc. (MA, USA)，secondary antibody to mouse/rabbit IgG was purchased from Nanjing Keygen Biotech Co. Ltd (Nanjing, China). StemSpan Serum-Free Expansion Medium (SFM) was purchased from STEMCELL Technologies Inc. (BC, Canada). Annexin-V-Fluos staining kit were purchased from Roche Diagnostics (IN, USA), DNA damage detect kit was purchased from BD corporation, ROS detection kit was purchased from Beyotime Company (Jiangsu, China). *N*-acetylcysteine (NAC), propidium iodide (PI), and all other chemicals were obtained from Sigma Chemical Co.

### Cell proliferation and viability assays

The cells were grown in 96-well plates in growth medium, and then treated with varying concentrations of XN4 for 48 h. After the treatment, the number of viable cells was measured using the MTT, colorimetric, dye-reduction method. The concentration of the drug at which cell growth was inhibited by 50% (IC_50_) was estimated using the GraphPad Prism5 software. All assays were performed in triplicate.

### Detection of intracellular ROS

Intracellular ROS was detected using an oxidation-sensitive fluorescent probe (DCFH-DA) [5]. After treatment with variety concentrations of XN4 for 12 h or 24 h, cells were washed twice，then incubated cells with 10 μM DCFH-DA at 37 °C for 20 min. DCFH-DA was deacetylated by nonspecific esterase intracellular, which was oxidized by ROS to the fluorescent compound 2, 7-dichlorofluorescein (DCF). DCF fluorescence was detected by FACScan cytometry. The intensity of DCF was recorded [14].

### Real-time quantitative reverse transcriptase-PCR (qRT- PCR)

K562 and K562/G01 cells were treated with either 6 μM XN4 alone, 5 mM NAC for 12 h, or 5 mM NAC for 1 h, followed by the addition of 6 μM XN4 for 12 h. The total cellular RNA was extracted using TRIzol (Invitrogen, CA, USA). First-strand cDNA was synthesized from 2 mg of total RNA using the Transcriptor First Strand cDNA Synthesis Kit (Roche, Shanghai, China), according to the manufacturer’s instructions. The levels of *Nox1*, *Nox2*, *Nox3*, *Nox4* and *Nox5* mRNA were determined by real-time PCR using SYBR Green I Master and a LightCycler 96 system (Roche). The changes in mRNA expression were calculated by the comparative Ct method: fold change = 2^-ΔΔC^_T_ = [(C_T_ gene of interest—C_T_ internal control)sample A—(C_T_ gene of interest—C_T_ internal control)sample B]. The experimental data were normalized to *GAPDH*. The primer sequences are listed in Table 1.

**Table 1:** The primer sequences.

Gene name	Access No	Forward / Reverse primer
**Nox1**	**NM_013955**	**GCAAATGCTGTCACCGATATTC/ TGCAGATTACCGTCCTTATTCC**
**Nox2**	**NM_000397**	**GCTATGAGGTGGTGATGTTAGT/ CTTCAGATTGGTGGCGTTATTG**
**Nox3**	**NM_015718**	**TGAGGGTCTCTCCACCATATT/ ACTCCTCCTCTTCATACCAGTAG**
**Nox4**	**NM_024505**	**ACCTCAACTGCAGCCTTATC/ ATCCAACAATCTCCTGGTTCTC**
**Nox5**	**NM_024505**	**CATCCAGTTCCACCAGCTTAT/ AGCCTGGAGTACAAAGTTCAC**
**GAPDH**	**NM_002046**	**CGGAGTCAACGGATTTGGTCGTAT/ AGCCTTCTCCATGGTGGTGAAGAC**

### DNA damage determination [15–16]

K562 and K562/G01 cells were collected, counted and diluted/concentrated to 5×10^5^ cells/ml, then plated in 24-well plates. They were treated with 6 μM XN4 for 12 h, 5 mM NAC for 12 h, or 5 mM NAC for 1 h, followed by 6 μM XN4 for 12 h. After the treatment, the cells were washed, suspended with 100 μl per tube of BD Cytofix/Cytoperm fixation and permeabilization solution, and incubated for 15 minutes on ice. The cells were washed again before being incubated with 20 μl of BD Perm/Wash Buffer, 5μl of an Alexa Fluor 647-conjugated mouse anti-H2AX (pS139) antibody, 5μl of an PerCP-Cy5.5-conjugated anti-BrdU antibody, and 5μl of a PE-conjugated anti-cleaved PARP (Asp214) antibody for 20 minutes at room temperature. The cells were washed once more before flow cytometry (Apoptosis, DNA damage and cell proliferation kit, Becton–Dickinson, NJ, USA).

### Neutral comet assay

Cells were collected after XN4 treatment for 12 h and processed for neutral comet assay using a CometAssay kit from Trevigen (Gaithersburg, MD) per the manufacturer’s protocol. Approximately 100 nucleus images were captured for each slide and processed by a Zeiss Axio Observer.Z1 microscope. The tail moments from the cells were measured by the TriTek CometScore software (Version 1.5.2.6; TriTek Corporation, VA).

### Cell cycle progression assay

K562 and K562/G01 cells were treated with 6 μM XN4 for 12 h, 5 mM NAC for 12 h, or 5 mM NAC for 1 h, followed by 6 μM XN4 for 12 h, before they were collected and fixed overnight in 70% ethanol at -20°C. After two washes with PBS, the cells were incubated with 1 μl of a propidium iodide solution (50 μg/ml) in darkness for 15 min at room temperature. The relative DNA content of these cells was analyzed by FACScan cytometry (Becton–Dickinson, NJ, USA) based on red fluorescence. The ModFit LT for Mac 3.0 software (Becton–Dickinson, NJ, USA) was used to quantify the fraction of the cell population in each cell cycle stage.

### Cell apoptosis examined by Annexin V-FITC and PI staining

K562 and K562/G01 cells in 24-well plates were incubated with 6 μM XN4 or 5 mM NAC for the indicated time, or with 5 mM NAC for 1 h before 6 μM XN4 was added for the indicated time. The cells were harvested and then incubated with 5 μl of Annexin V-FITC and 5 μl of PI, at 37 ° C in the dark for 15 min. The samples were transferred to FCM tubes to measure cell fluorescence. During early apoptosis, cells become Annexin-V positive, but as apoptosis progresses, they become positive for both Annexin-V and PI.

### Immunoblotting analyses

After indicated treatment of XN4, cells were harvested and washed twice with ice-cold PBS. Cell extracts were collected in cell lysis buffer (2% Nonidet P-40, 0.5% sodium deoxycholate, 0.1% SDS, 100 μg/mL Phenylmethylsulfonyl fluoride, 10 μg/mL aprotinin, 10 μg/mL leupeptin). Equal amounts of lysate protein from various treatments was resolved by SDS-PAGE and transferred onto PVDF membrane. After blocking with 5% non-fat milk in TBST buffer (10 mmol/L Tris-HCl, 150 mmol/L NaCl, and 0.1% Tween 20, pH 8.0) for 2 h at room temperature, the membranes were incubated overnight at 4 ℃ with primary monoclonal antibody at 1:1000 dilution. Then the membranes were incubated for 2 h with horseradish peroxidase-conjugated secondary antibody (1:1000 dilutions). After washing thrice with TBST buffer, the protein-antibody complex detection was performed using enhanced chemiluminescence reagent and visualized in an image capture software Image station system 4000MM.

### Analysis of CD34+ leukemia progenitor/stem cell apoptosis

Bone marrow samples were obtained with written informed consent from three patients with CML. This study was approved by the Ethical Review Committee for Biomedical Research of Fujian Medical University. Mononuclear cells from CML bone marrow were treated with or without XN4 for 48 h. The cells were labeled with CD34-PE-Cy7 and CD38-PE (eBioscience Inc, CA, USA) and then analyzed by flow cytometry for apoptosis via Annexin V-APC staining.

### Statistical analysis

The group differences were examined using an unpaired Student’s t test. Differences were considered significant at *p<0*.05. All of the analyses were performed using the SPSS (Statistical Package for the Social Sciences) software.

## Results

### XN4 inhibits CML cell proliferation

To investigate the biological effects of XN4, K562 and K562/G01 cells were treated with various doses of XN4 for 48 h and the cells’ viability was assayed. K562 is a human blast CML cell line and is sensitive to imatinib. The resistant cell line, K562/G01, was established with more than 30-fold resistance to imatinib compared to its sensitive parental cell line. Amplification of the *bcr-abl* gene in K562/G01 cells was observed by FISH staining [17]. When K562 or K562/G01 cells were exposed to XN4 at various concentrations for 48 h, cell growth was inhibited in a concentration-dependent manner (Fig 1B). In addition, there was no significant difference between the imatinib-sensitive K562 and imatinib-resistant K562/G01 cells in their sensitivity to XN4. The IC_50_ values for the K562 and K562/G01 cell lines were 3.75±0.07 μM and 2.63±0.43 μM, respectively. Thus, the activity of XN4 is approximately 100-fold higher than its parental compound Nov (Fig 1B).

### Effects of XN4 on the intracellular ROS level in CML cells

**Fig 2:**
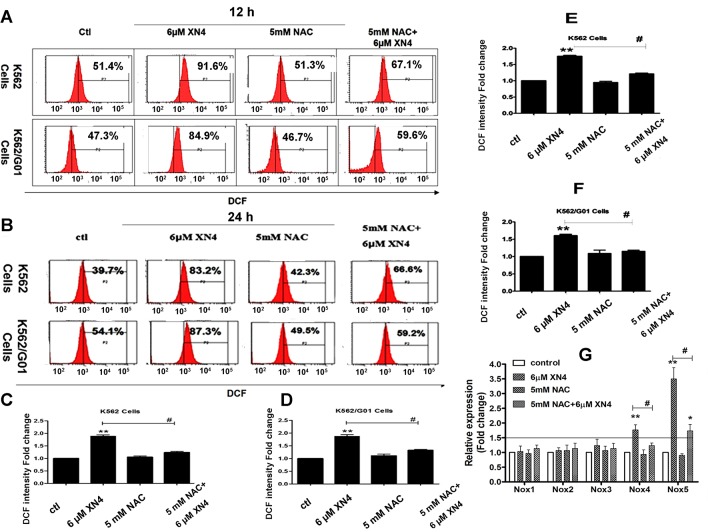
Effects of XN4 on intracellular ROS levels and expression of *Nox* genes in CML cells. The fluorescence of DCF shifted after XN4 treatment for 12 h (A, C, D) or 24 h (B, E, F), with or without 5 mM NAC pre-treatment for 1 h. Quantification of DCF intensity fold-change after XN4 treatment. All data are expressed as the mean±SD. (** *p<*0.01 vs control group, # *p<*0.01 XN4 group vs NAC+XN4 group, n = 3). (G) The levels of *Nox1*, *Nox2*, *Nox3*, *Nox4*, and *Nox5* mRNA were determined using real-time quantitative RT-PCR. The data are presented as fold-changes, which are normalized to *GAPDH* expression and expressed as the mean±SD. (** *p<*0.01 vs control group, # *p<*0.01 XN4 group vs NAC+XN4 group, n = 3).

We hypothesized that the XN4 anti-tumor activity could derive from the activation of ROS. To investigate the XN4 mechanism, we applied DCFH-DA to detect the level of intracellular ROS. Observation of both K562 and K562/G01 cells showed a concentration-dependent increase in intracellular ROS after treatment with different concentrations of XN4 for 12 h or 24 h, compared to the untreated control group. However, after pre-treatment with the antioxidant NAC (5 mM) for 1 h, ROS generation was significantly reduced (Fig 2A–2F). For K562 and K562/G01 cells, XN4 significantly increased intracellular ROS levels compared to the control group (** *p<*0.01, n = 3). 5 mM NAC did not influence the intracellular ROS of K562 and K562/G01 cells. Notably, the 1 h NAC pre-treatment clearly attenuated the changes in ROS after XN4 treatment (# *p<*0.01, n = 3, Fig 2A–2F).

### Effects of XN4 on mRNA expression of *Nox* gene family

We sought to determine the mechanisms by which XN4 induces ROS generation in CML cells. It has been reported that the NADPH oxidases (Noxs) play an important role in generating superoxide and other ROS. We investigated the mRNA levels of the *Nox1*, *Nox2*, *Nox3*, *Nox4* and *Nox5* genes in CML cells by real-time quantitative PCR. The data indicate that 6 μM XN4 treatment selectively increases *Nox4* and *Nox5* expression (** *p<*0.01, n = 3) in both K562 and K562/G01 cells (Fig 2G) and that the 1 h 5 mM NAC pretreatment significantly attenuated the increase in *Nox4* and *Nox5* mRNA induced by XN4 (# *p<*0.01, n = 3). Together, these data suggest that increased *Nox4* and *Nox5* expression are linked to XN4-induced ROS generation in CML cells.

### XN4 induces DNA double-strand breaks (DSBs) in CML cells

**Fig 3:**
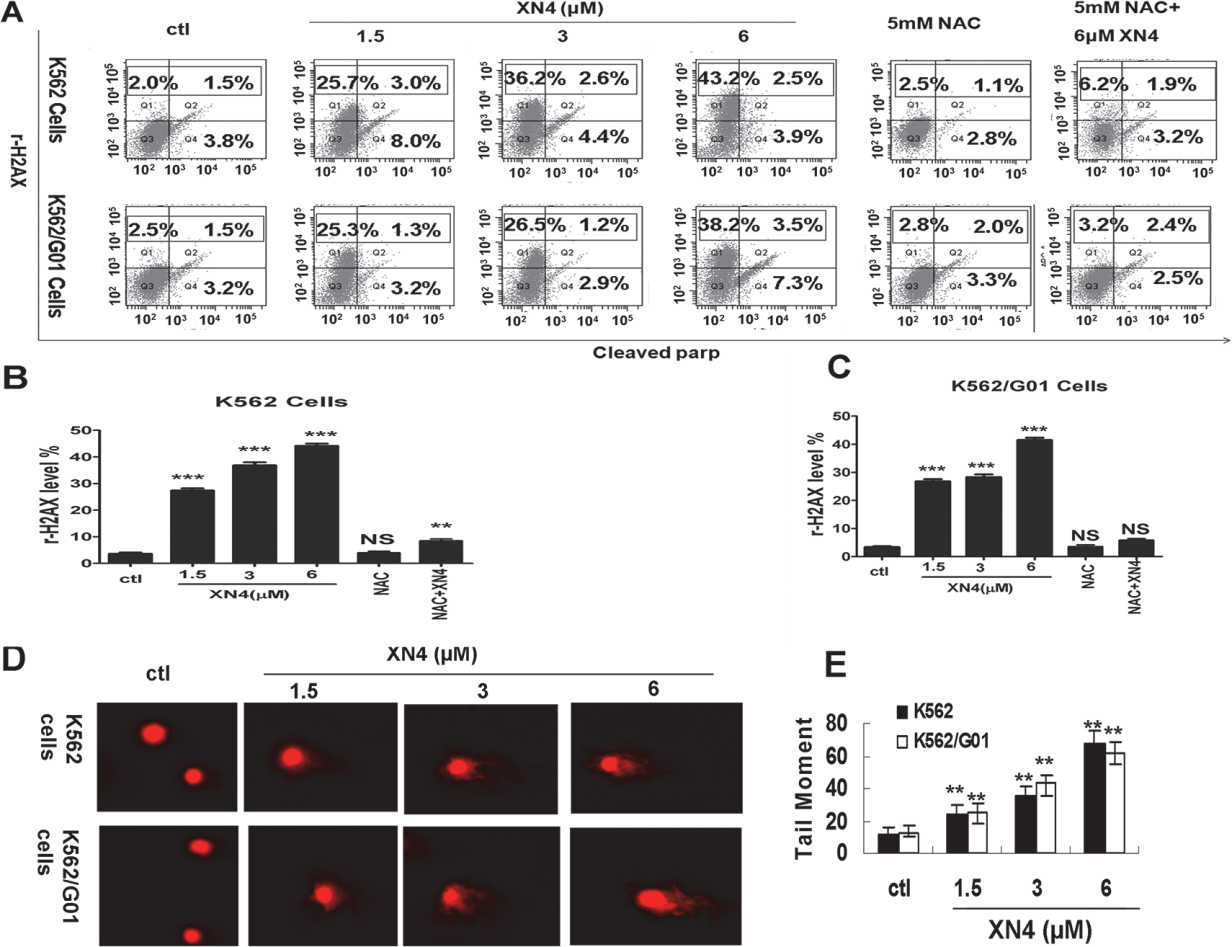
The effect of XN4 on DSBs of DNA damage. (A) Representation of the fluorescence shift of rH2AX and cleaved PARP. K562 and K562/G01 cells were treated with XN4 for 12 h, 5 mM NAC for 12 h, or 5 mM NAC for 1 h followed by 6 μM XN4 for 12 h. After the treatment, the cells were washed, and then incubated with Alexa Fluor 647-conjugated mouse anti-H2AX (pS139) antibody and 5μl of a PE-conjugated anti-cleaved PARP (Asp214) antibody for 20 minutes at room temperature. The cells were washed once more before flow cytometry. (B, C) Quantification of rH2AX-positive ratio. The data are expressed as the mean±SD (*** *p<*0.001, ** *p<*0.01 vs control group, *p* = NS no significance, n = 3). (D) Representation of DSBs by neutral comet assay. Cells were collected after XN4 treatment for 12 h and processed for neutral comet assay using a CometAssay kit. Approximately 100 nucleus images were captured for each slide and processed by a Zeiss Axio Observer.Z1 microscope. The tail moments from the cells were measured by the TriTek CometScore software. (E) Quantification of the average tail moments (mean±SD) from three independent experiments are presented (** *p<*0.01 vs control group).

It is well known that the production of ROS is a destructive aspect of oxidative stress that can cause DNA damage [5–8]. DNA double-strand breaks (DSBs) are considered the most detrimental DNA damage and a single unrepaired DSB is sufficient to kill a cell. Following the induction of DNA DSBs, H2AX is phosphorylated at Ser139 (rH2AX) and collaborates with many other proteins to mediate important biological functions in cells. To test whether XN4 causes DNA DSBs in K562 cells, we measured rH2AX, after treating with 6 μM XN4 for 12 h, as a surrogate marker of DSBs. The results revealed that XN4 could increase the proportion of rH2AX dramatically (** *p<*0.01, n = 3). In contrast, after pre-treatment with NAC (5 mM) for 1 h, the DNA damage was significantly restored (# *p<*0.01, n = 3). In parallel, cleaved PARP was also increased, which also indicates apoptosis induction resulting from DNA DSBs (Fig 3A–3C). Because phosphorylation of H2AX signals to the repair machinery rapidly, it does not reflect true DNA breaks. In order to further verify DNA breaks we performed single cell gel-electrophoresis (Neutral comet assay) which is a more powerful and direct assay for the determination of DNA breaks. Cells were collected after XN4 treatment for 12 h and processed for neutral comet assay using a CometAssay kit. This result revealed that XN4 could increase the tail moment of K562 and K562/G01 cells dramatically (** *p<*0.01, n = 3) in a concentration-dependent manner (Fig 3D and 3E).

**Fig 4:**
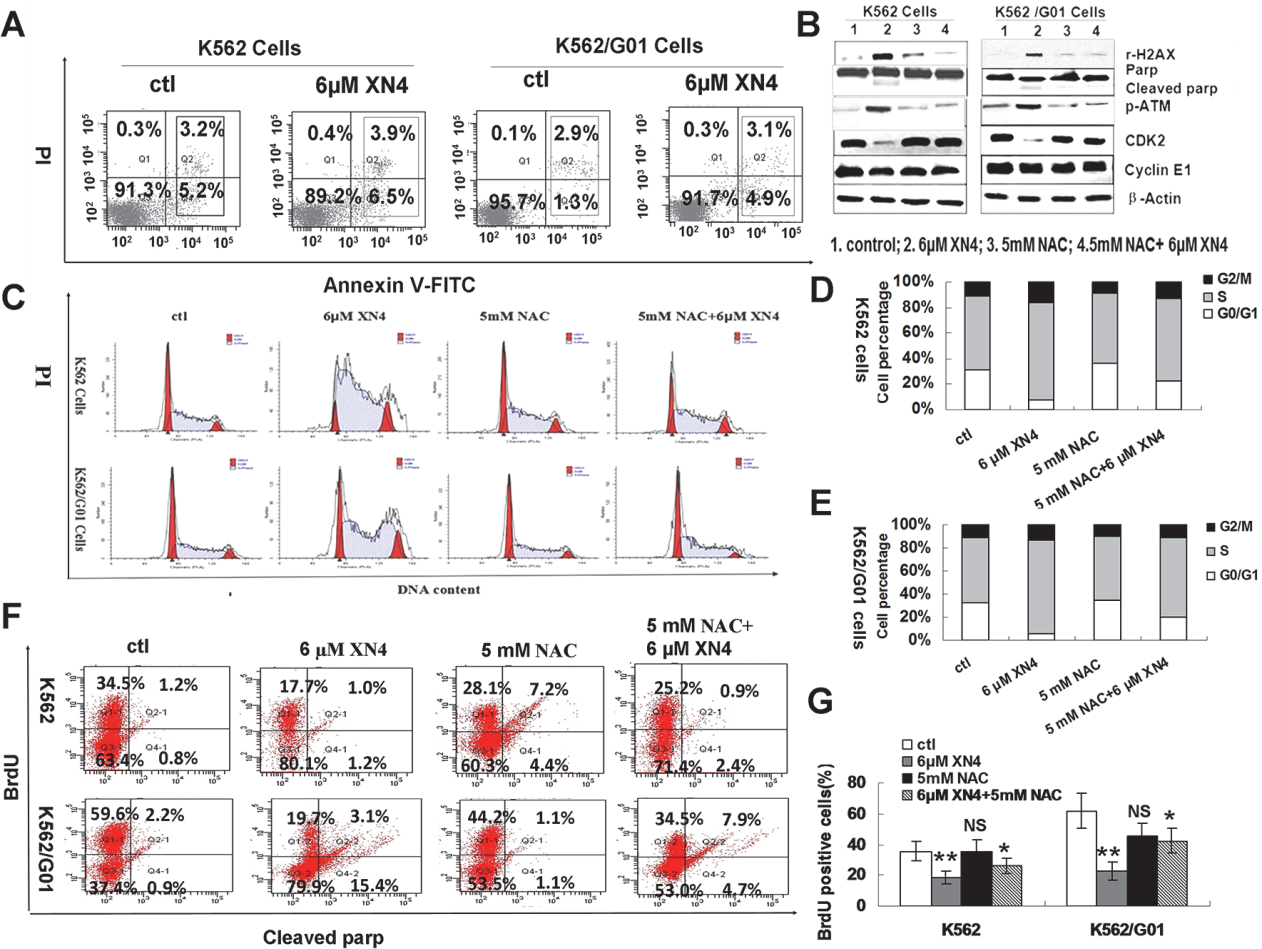
Effects of XN4 on cell cycle arrest in CML cells. (A) Representation of apoptosis ratio after XN4 treatment for 12 h. (B) Effects of XN4 on the activation of DNA-damage-sensing kinases in CML cells. K562 and K562/G01 cells were treated with 6 μM XN4 for 12 h, with or without NAC pre-treatment. The data demonstrate that XN4 increased the phosphorylation level of rH2AX and ATM, but decreased CDK2 and CyclinE1; β-Actin served as the protein-loading control. 1. control; 2. XN4 6 μM; 3. NAC 5 mM; 4. NAC 5 mM+XN4 6 μM. (C-E) K562 and K562/G01 cells were cultured in the presence of XN4 for 48 h, with or without pre-treatment with 5 mM NAC for 1 h, harvested, and then stained with PI and subsequently analyzed by flow cytometry with quantitation using the FlowJo software. (F) Representation of the fluorescence shift of BrdU after XN4 treatment, with or without NAC pre-treatment. K562 and K562/G01 cells were treated with 6 μM XN4 for 12 h, 5 mM NAC for 12 h, or 5 mM NAC for 1 h followed by 6 μM XN4 for 12 h. After the treatment, the cells were labeled with BrdU for 30 min, washed, and then incubated with 5μl of an PerCP-Cy5.5-conjugated anti-BrdU antibody for 20 minutes at room temperature. The cells were washed once more before flow cytometry (G) Quantification of the fluorescence shift of BrdU The data are expressed as the mean±SD. (** *p<*0.01, * *p<*0.05 vs control group, *p* = NS no significance, n = 3).

Additionally, to exclude the possibility that this DNA damage is due to apoptosis, apoptosis at this time point was detected. In contrast to the obvious effect of DNA damage, XN4 caused almost no induction of apoptosis in K562 and K562/G01 cells at this time point. The proportion of Annexin-V positive cells, which is a marker of early apoptosis, is comparable to that of the control group (Fig 4A).

### The effects of XN4 on the activation of DNA-damage- sensing kinases in CML cells

To confirm the DNA damage induced by XN4 in K562 and K562/G01 cells, DNA-damage-sensing kinases were detected. As Fig 4B shows, XN4 increased the levels of rH2AX and ATM, but decreased the level of CDK2 dramatically. Moreover, NAC attenuated the alteration induced by XN4. These results demonstrate that XN4 can cause the lethal DSB and activate DNA damage signaling through ROS generation in CML cells.

### DNA damage–induced cell-cycle arrest by XN4 in CML cells

The cell cycle checkpoint is activated by various types of external or internal stimuli that induce DNA damage, thus integrating DNA repair with cell cycle progression [18–20]. Treatment of K562 and K562/G01 cells with 6 μM XN4 for 48 h caused cell cycle arrest in S and G2/M phase (Fig 4C). The percentage of cells in S and G2/M phase increased significantly, compared to the control group, but pre-treatment with 5 mM NAC for 1 h decreased the number of cells arrested in G2/M phase, in both K562 and K562/G01 cells (Fig 4C–4E). To show conclusively that there is no proliferation and DNA replication is stalled despite increase of cells in S phase. BrdU incorporation was performed. Treatment of K562 and K562/G01 cells with 6 μM XN4 for 48 h inhibited BrdU incorporation significantly. In contrast, after pre-treatment with NAC (5 mM) for 1 h, the BrdU incorporation was restored (Fig 4F and 4G).

### XN4 induces apoptosis of CML cells after DNA damage

**Fig 5:**
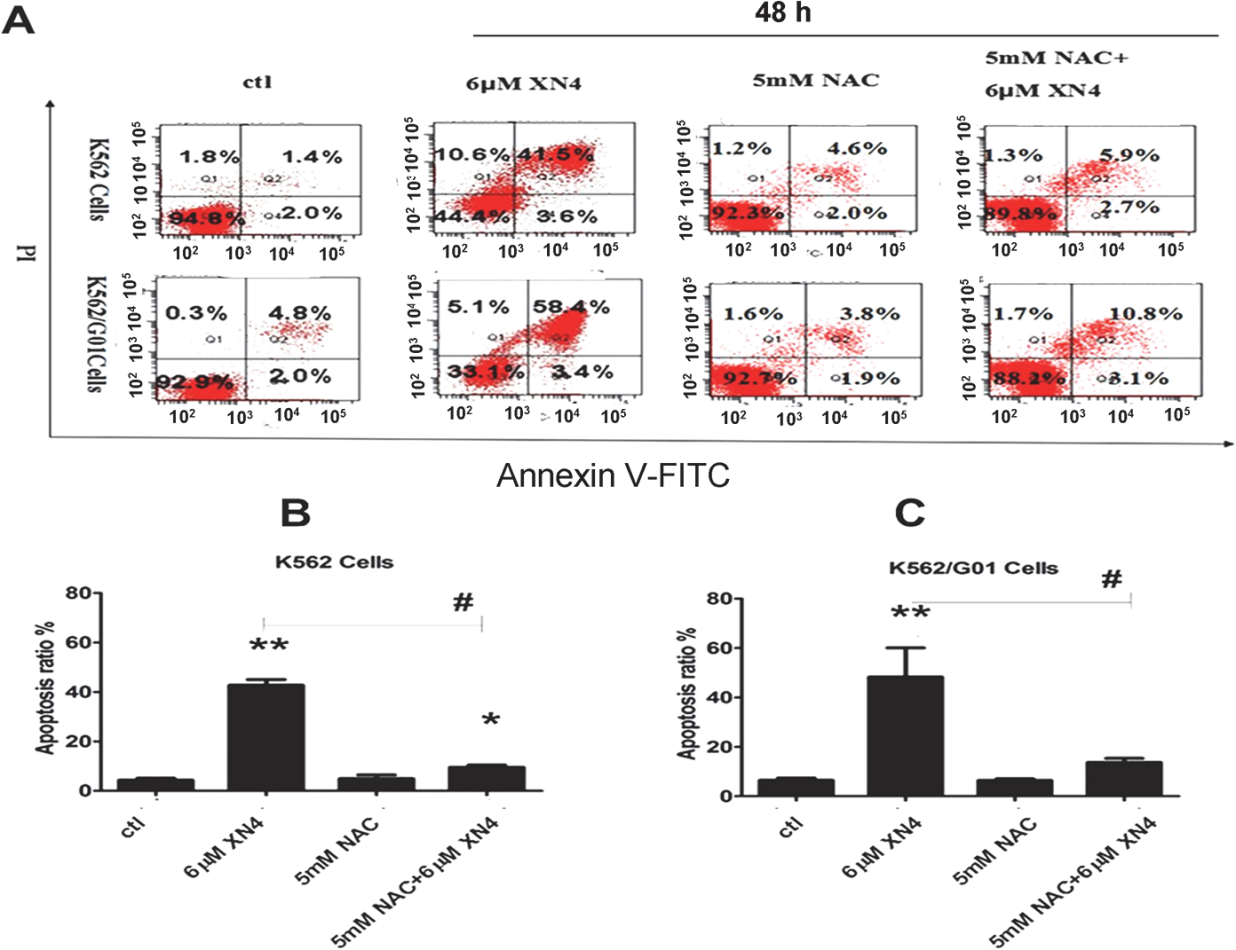
Effects of XN4 on apoptosis induction in CML cells. K562 and K562/G01 cells were incubated 6 μM XN4 or 5 mM NAC for 48 h, and 5 mM NAC pre-treated for 1 h then added 6μM XN4 for 48 h. The cells were harvested, then double-stained with PI and Annexin V-FITC, and subsequently analyzed by flow cytometry. All tests were performed three times, and the data are expressed as the mean±SD (** *p<*0.01, * *p<*0.05 vs control group, # *p<*0.01 XN4 group vs NAC+XN4 group, n = 3).

Following the induction of DNA damage, a prominent route of cellular inactivation is apoptosis, thus we speculated that apoptosis should be induced by XN4. As expected, treatment with 6 μM XN4 for 48 h significantly increased the proportion of apoptotic cells, in both K562 and K562/G01 cells (** *p<*0.01, n = 3). Just as NAC pre-treatment successfully ablated the XN4-mediated ROS generation and DNA damage, the antioxidant prevented XN4-induced apoptosis (# *p<*0.01, n = 3) (Fig 5A–5C). Thus, our results indicate that NAC, an antioxidant, diminishes the DNA-damaging effects of XN4 in CML cells, in both imatinib-sensitive and imatinib-resistant cells, which confirms that XN4 damages DNA through oxidative stress.

### XN4 potently inhibits the growth of human leukemia progenitor/stem cells

**Fig 6:**
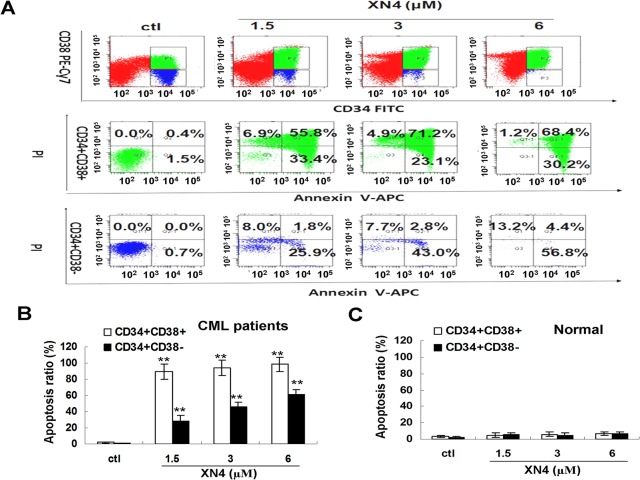
XN4 induces apoptosis in CML or normal primitive and committed progenitors *in vitro*. (A) XN4 induced apoptosis in CML CD34^+^CD38^—^primitive and CML CD34^+^CD38^+^-committed progenitors. CML CD34^+^CD38^-^ primitive and CD34^+^CD38^+^ committed progenitors were exposed to XN4 at the indicated concentrations for 48 h. (B) Apoptosis in CML primitive and committed progenitors was analyzed by FACS as the percentage of cells positively labeled with Annexin V-APC (** *p<*0.01 vs control group, n = 3). (C) Apoptosis in normal primitive and committed progenitors from normal bone marrow was analyzed by FACS as the percentage of cells positively labeled with Annexin V-APC (n = 3).

To determine whether these events could be extended to primary leukemia progenitor/stem cells from patients, the CD34+CD38+ and the more primitive CD34+CD38- fractions were analyzed to determine their apoptosis levels after being cultured in SFM alone. XN4 significantly induced apoptosis in the CD34+CD38+ and CD34+CD38- fractions (Fig 6), highlighting the activity of this drug in both quiescent and cycling cell populations. After they were treated with 1.5 μM XN4, the proportions of CD34+CD38+ and CD34+CD38- cells in apoptosis were 89.2% and 27.7%, respectively. In addition, XN4 is somewhat selective between normal and CML stem cells (Fig 6C).

## Discussion

Our experiments verified that not only is the activity of XN4 much higher than Nov, XN4 potently inhibits the proliferation of K562 and K562/G01 cells. Additionally, we observed that XN4 causes oxidative stress, resulting in the generation of ROS. Many studies have shown that Noxs play important roles in the generation of ROS [21]. ROS generation contributes to the anti-cancer activities of several cancer therapies. Thus, the manipulation of ROS generation in cancer cells is a potential therapeutic strategy [22–25]. In this study, we found that treatment with XN4 greatly increases the expression of *Nox4* and *Nox5* before increasing ROS production in CML cells, which indicates that XN4 may induce ROS generation, at least partially, by increasing *Nox4* and *Nox5* expression. It is well known that increased oxidative stress causes DNA damage followed by cell cycle arrest in cancer cells.

Almost all types of cells, but especially the tumor cells harboring an intrinsic capability in response to DNA damage, will attempt to repair non-lethal damage by applying a variety of damage repair pathways, for example, base excision repair, homologous recombination repair, non-homologous end joining repair and mismatch repair [26–28]. rH2AX is a sensitive biomarker of DNA damage [29–30]. It accumulates at the site of DNA breakage, and then recruits other proteins related to DNA damage and repair, for example ATM, Mre11, and NBS1 [31–32]. In this study, we assessed DSBs by three approaches, Western blot and FCM examination of rH2AX, and neutral comet assay. All assays provided clear evidence of DSB induction by XN4 treatment; however, quenching ROS with NAC diminished XN4-mediated DSB formation in CML cells.

Additionally, DNA damage can activate the cell cycle check point, resulting in cell cycle arrest to allow time to repair the damage before it is replicated. PARP plays an important role in DNA damage repair, but it is cleaved and inactivated in apoptosis [33–35]. In our test, we observed that XN4 can increase the levels of rH2AX and PARP cleavage, which demonstrates that XN4 induces serious DNA damage and DNA damage repair failure. Although the cell cycle arrested in S phase, which should provide enough time to perform damage repairs, the cells still entered apoptosis.

Furthermore, the accumulation of LSCs (leukemia stem cells) plays a critical role in CML relapse after imatinib therapy [12, 36]. Importantly, TKIs effectively target proliferating mature cells but do not eradicate quiescent LSCs, thereby allowing the disease to persist, despite treatment. Accordingly, it is essential to develop alternative strategies that target the LSCs population. XN4 can induce apoptosis in both quiescent (CD34+CD38-) and cycling (CD34+CD38+) cell populations, which indicate that XN4 might target the LSCs population.

In conclusion, XN4 induced ROS formation, led to DNA damage, and increased the number of cells in S and G2/M phase. On the other hand, the XN4-mediated accumulation of ROS triggered cell apoptosis. Thus, this study suggests that XN4 has the potential to be developed into an effective anti-cancer agent.
